# (*S*)-(−)-2-(1*H*-Indol-3-yl)-*N*-(1-phenyl­eth­yl)acetamide

**DOI:** 10.1107/S1600536812028450

**Published:** 2012-06-30

**Authors:** Johana Ramírez, Oscar Romero, Jorge R. Juárez, Joel L. Terán, Angel Mendoza

**Affiliations:** a Centro de Química, Instituto de Ciencias, Benemérita Universidad Autónoma de Puebla, 72570, Puebla, Pue., Mexico

## Abstract

In the title compound, C_18_H_18_N_2_O, the dihedral angle between the indole system and the phenyl ring is 17.2 (2)°. The crystal packing features two N—H⋯O hydrogen bonds, which link the mol­ecules into layers parallel to (001). The absolute configuration was determined by the synthetic procedure and was set according to the starting material.

## Related literature

For background to the synthesis of chiral non-racemic acetamide indole compounds, see: Kochanowska-Karamyan & Hamann (2010 ▶). For their use in the synthesis of nitro­gen heterocyclic compounds and indole alkaloids, see: Suárez-Castillo *et al.* (2006 ▶); Chiou *et al.* (2009 ▶).
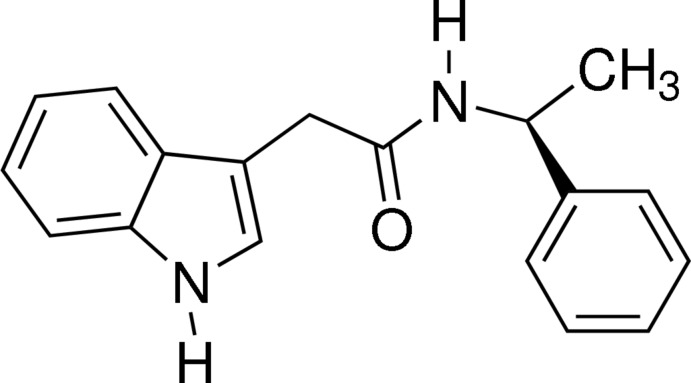


## Experimental

### 

#### Crystal data

C_18_H_18_N_2_O
*M* *_r_* = 278.34
Orthorhombic, 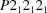

*a* = 7.307 (4) Å
*b* = 8.559 (4) Å
*c* = 25.674 (9) Å
*V* = 1605.7 (13) Å^3^
*Z* = 4
Mo *K*α radiation
μ = 0.07 mm^−1^
*T* = 298 K
0.65 × 0.6 × 0.1 mm

#### Data collection

Siemens P4 diffractometer
Absorption correction: ψ scan (North *et al.*, 1968 ▶) *T*_min_ = 0.646, *T*_max_ = 1
2937 measured reflections
2126 independent reflections
1146 reflections with *I* > 2σ(*I*)
*R*_int_ = 0.045
3 standard reflections every 97 reflections intensity decay: 1%

#### Refinement

*R*[*F* ^2^ > 2σ(*F* ^2^)] = 0.065
*wR*(*F* ^2^) = 0.161
*S* = 1.05
2126 reflections
199 parameters
H atoms treated by a mixture of independent and constrained refinement
Δρ_max_ = 0.15 e Å^−3^
Δρ_min_ = −0.15 e Å^−3^

### 

Data collection: *XSCANS* (Siemens, 1994 ▶); cell refinement: *XSCANS*; data reduction: *XSCANS*; program(s) used to solve structure: *SHELXS97* (Sheldrick, 2008 ▶); program(s) used to refine structure: *SHELXL97* (Sheldrick, 2008 ▶); molecular graphics: *ORTEP-3* (Farrugia, 1997 ▶); software used to prepare material for publication: *SHELXL97*.

## Supplementary Material

Crystal structure: contains datablock(s) global, I. DOI: 10.1107/S1600536812028450/bt5951sup1.cif

Structure factors: contains datablock(s) I. DOI: 10.1107/S1600536812028450/bt5951Isup2.hkl

Supplementary material file. DOI: 10.1107/S1600536812028450/bt5951Isup3.cml

Additional supplementary materials:  crystallographic information; 3D view; checkCIF report

## Figures and Tables

**Table 1:** Hydrogen-bond geometry (Å, °)

*D*—H⋯*A*	*D*—H	H⋯*A*	*D*⋯*A*	*D*—H⋯*A*
N1—H1*N*⋯O1^i^	0.89 (5)	2.03 (5)	2.891 (4)	163 (4)
N2—H2*N*⋯O1^ii^	0.96 (6)	1.91 (6)	2.847 (5)	164 (4)

Symmetry codes: (i) 
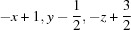
; (ii) 
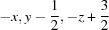
.

**Fig. 1.**
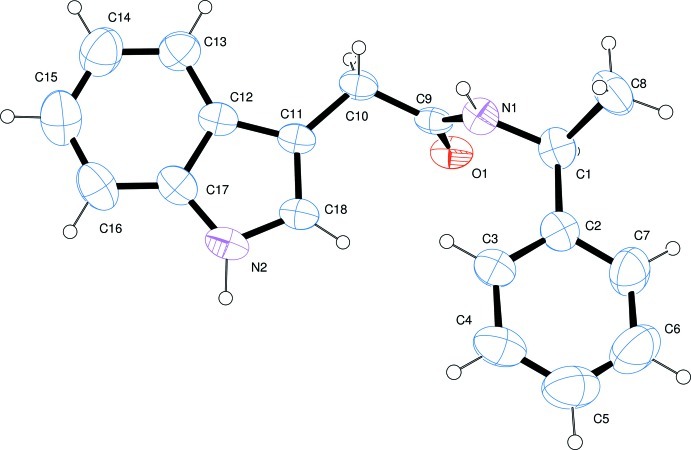
The molecular structure of title compound, with atom labels and 30% probability displacement ellipsoids for non-H atoms.

**Fig. 2.**
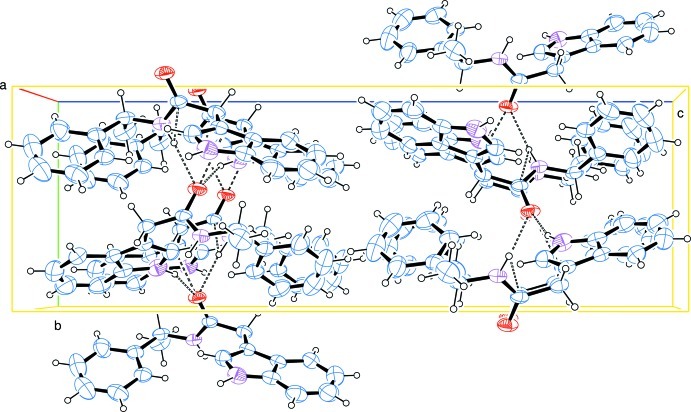
Molecular packing of title compound, viewed down the a axis, showing hydrogen bonds and intramolecular interaction (dashed lines).

## supplementary crystallographic information

### Comment

The synthesis of chiral non racemic acetamides indole compounds is an original area of interest in organic chemistry (Kochanowska-Karamyan & Hamann, 2010) because they are useful intermediates for the synthesis of diverse interesting nitrogen heterocyclic compounds and indole alkaloids derivatives and natural products (Suárez-Castillo *et al.*, 2006; Chiou *et al.*, 2009).

In the title compound the N1 atom in a planar conformation from the plane between C9, H1N and C1 with an r.m.s. deviation of 0.012Å. The N1—C9 [1.322 (5) Å] and C9—O1 [1.254 (4) Å] distances show electron delocalization along the N1—C9—O1 system. The indole group shows a torsion angle of 102.1 (4)° from the plane placed by N1/C9/C10/C11 and phenyl ring C2—C7 shows a torsion angle of 92.3 (4)° from plane C9/N1/C1/C2. The crystal packing is stabilized by two hydrogen bond interactions [N1—H1N···O1 and N2—H2N···O1] linking the molecules into planes parallel to (0 0 1) (Table 1).

### Experimental

The title compound, C_18_H_18_N_2_O, was obtained dissolving indolic acid (0.96 mmol, 0.277 g) and boric acid (0.30 mmol, 0.020 g) in 88 ml of toluene under N_2_ atmosphere. Once the mixture was colorless, (*S*)-(-)-phenylethylamine was added and heated under reflux by 16 h. After that, mixture was cooled and hexane (0.5 *L*) was added. Finally, white solid was obtained and crystalized from an ethyl acetate/diethylether solution; m. p. 94–96 °C. [α]_D_^25^ = -45.7° (*c* 1.5, CH_2_Cl_2_); IR (KBr)1643 cm^-1. 1^H NMR (CDCl_3_, 400 MHz) *δ* (p.p.m.), *J*(Hz) 9.24 (s, 1H), 7.48 (d, *J*=8.0, 1H),7.26 (d, *J*=8.4, 1H), 7.17–7.05 (m, 6H),6.85 (d, *J*=2.4, 1H), 6.32 (d, *J*=8.0, 1H), 5.12 (m, 1H), 3.69 (d, *J*=3.2, 2H), 1.24 (d, *J*=7.2, 3H). ^13^C NMR (CDCl_3_,100 MHz) *δ* (p.p.m.) 22.2, 33.8, 49.1, 108.2,112.1, 118.8–128.8, 136.9, 143.4, 171.8.

### Refinement

H atom bonded to N atoms were located in a difference Fourier map and they were isotropically refined. H atoms bonded to C atoms were placed in geometrical idealized positions and refined as riding on their parent atoms, with C—H = 0.93–0.98 Å and with *U*_iso_(H) = 1.2 *U*_eq_(C) or *U*_eq_(H) = 1.5 *U*_eq_(C) for methyl groups. In the absence of anomalous scatterers, the absolute configuration could not be determined. It was set according to the starting material and Friedel pairs were merged.

### Crystal data

**Table:**

C_18_H_18_N_2_O	*F*(000) = 592
*M**_r_* = 278.34	*D*_x_ = 1.151 Mg m^−^^3^
Orthorhombic, *P*2_1_2_1_2_1_	Mo *K*α radiation, λ = 0.71073 Å
Hall symbol: P 2ac 2ab	Cell parameters from 24 reflections
*a* = 7.307 (4) Å	θ = 12.6–22.7°
*b* = 8.559 (4) Å	µ = 0.07 mm^−^^1^
*c* = 25.674 (9) Å	*T* = 298 K
*V* = 1605.7 (13) Å^3^	Prism, colourless
*Z* = 4	0.65 × 0.6 × 0.1 mm

### Data collection

**Table:**

Siemens P4 diffractometer	*R*_int_ = 0.045
Graphite monochromator	θ_max_ = 27.5°, θ_min_ = 2.5°
2θ/ω scans	*h* = −2→9
Absorption correction: ψ scan (North *et al.*, 1968)	*k* = −4→11
*T*_min_ = 0.646, *T*_max_ = 1	*l* = −11→33
2937 measured reflections	3 standard reflections every 97 reflections
2126 independent reflections	intensity decay: 1%
1146 reflections with *I* > 2σ(*I*)	

### Refinement

**Table:**

Refinement on *F*^2^	Primary atom site location: structure-invariant direct methods
Least-squares matrix: full	Secondary atom site location: difference Fourier map
*R*[*F*^2^ > 2σ(*F*^2^)] = 0.065	Hydrogen site location: inferred from neighbouring sites
*wR*(*F*^2^) = 0.161	H atoms treated by a mixture of independent and constrained refinement
*S* = 1.05	*w* = 1/[σ^2^(*F*_o_^2^) + (0.0706*P*)^2^ + 0.0614*P*] where *P* = (*F*_o_^2^ + 2*F*_c_^2^)/3
2126 reflections	(Δ/σ)_max_ < 0.001
199 parameters	Δρ_max_ = 0.15 e Å^−^^3^
0 restraints	Δρ_min_ = −0.15 e Å^−^^3^

### Special details

**Table:**

Geometry. All s.u.'s (except the s.u. in the dihedral angle between two l.s. planes) are estimated using the full covariance matrix. The cell s.u.'s are taken into account individually in the estimation of s.u.'s in distances, angles and torsion angles; correlations between s.u.'s in cell parameters are only used when they are defined by crystal symmetry. An approximate (isotropic) treatment of cell s.u.'s is used for estimating s.u.'s involving l.s. planes.
Refinement. Refinement of *F*^2^ against ALL reflections. The weighted *R*-factor *wR* and goodness of fit *S* are based on *F*^2^, conventional *R*-factors *R* are based on *F*, with *F* set to zero for negative *F*^2^. The threshold expression of *F*^2^ > 2σ(*F*^2^) is used only for calculating *R*-factors(gt) *etc*. and is not relevant to the choice of reflections for refinement. *R*-factors based on *F*^2^ are statistically about twice as large as those based on *F*, and *R*- factors based on ALL data will be even larger.

### Fractional atomic coordinates and isotropic or equivalent isotropic displacement parameters (Å^2^)

**Table:**

	*x*	*y*	*z*	*U*_iso_*/*U*_eq_	
O1	0.3536 (4)	1.0472 (3)	0.73306 (10)	0.0732 (8)	
C9	0.4310 (5)	0.9265 (4)	0.74986 (14)	0.0574 (9)	
N1	0.5307 (5)	0.8365 (4)	0.71908 (12)	0.0630 (8)	
C2	0.4248 (5)	0.7787 (4)	0.62951 (15)	0.0702 (11)	
N2	−0.0448 (5)	0.6926 (4)	0.81406 (16)	0.0822 (10)	
C1	0.5641 (6)	0.8626 (4)	0.66407 (15)	0.0712 (11)	
H1	0.5511	0.9749	0.6576	0.085*	
C3	0.2953 (5)	0.6773 (5)	0.64922 (16)	0.0714 (11)	
H3	0.2874	0.6622	0.685	0.086*	
C12	0.1826 (5)	0.7462 (4)	0.86932 (15)	0.0647 (10)	
C11	0.2352 (5)	0.8019 (4)	0.81856 (14)	0.0594 (9)	
C10	0.4134 (5)	0.8780 (4)	0.80602 (14)	0.0636 (10)	
H10A	0.5117	0.8061	0.8144	0.076*	
H10B	0.4278	0.9696	0.8279	0.076*	
C17	0.0087 (6)	0.6788 (4)	0.86442 (18)	0.0752 (11)	
C18	0.0937 (5)	0.7666 (5)	0.78644 (16)	0.0735 (11)	
H18	0.0899	0.7887	0.751	0.088*	
C4	0.1767 (7)	0.5977 (6)	0.6164 (2)	0.0978 (15)	
H4	0.091	0.5291	0.6303	0.117*	
C16	−0.0813 (7)	0.6093 (6)	0.9064 (2)	0.0980 (15)	
H16	−0.1963	0.5641	0.9025	0.118*	
C13	0.2681 (7)	0.7464 (5)	0.91787 (17)	0.0867 (13)	
H13	0.3831	0.791	0.9223	0.104*	
C7	0.4333 (8)	0.7996 (6)	0.57627 (19)	0.1064 (17)	
H7	0.5194	0.867	0.5619	0.128*	
C8	0.7594 (6)	0.8189 (6)	0.6517 (2)	0.0986 (15)	
H8A	0.7763	0.7089	0.6572	0.148*	
H8B	0.8407	0.8761	0.6741	0.148*	
H8C	0.7856	0.8439	0.616	0.148*	
C15	0.0072 (10)	0.6104 (7)	0.9535 (2)	0.121 (2)	
H15	−0.0488	0.5643	0.9822	0.146*	
C14	0.1770 (10)	0.6781 (7)	0.9594 (2)	0.1179 (18)	
H14	0.2318	0.678	0.9921	0.141*	
C6	0.3115 (11)	0.7186 (9)	0.5442 (2)	0.138 (2)	
H6	0.3174	0.7333	0.5084	0.166*	
C5	0.1852 (9)	0.6192 (9)	0.5639 (3)	0.127 (2)	
H5	0.1053	0.5664	0.5419	0.153*	
H1N	0.589 (6)	0.757 (6)	0.7342 (17)	0.104 (16)*	
H2N	−0.147 (8)	0.659 (6)	0.7935 (18)	0.130 (18)*	

### Atomic displacement parameters (Å^2^)

**Table:**

	*U*^11^	*U*^22^	*U*^33^	*U*^12^	*U*^13^	*U*^23^
O1	0.0751 (18)	0.0429 (13)	0.1016 (18)	0.0091 (14)	−0.0245 (16)	0.0040 (13)
C9	0.051 (2)	0.0402 (18)	0.080 (2)	−0.0085 (19)	−0.018 (2)	−0.0014 (17)
N1	0.072 (2)	0.0403 (16)	0.0771 (19)	0.0031 (17)	−0.0018 (18)	0.0044 (15)
C2	0.071 (3)	0.057 (2)	0.083 (3)	0.021 (2)	0.006 (2)	−0.0071 (19)
N2	0.060 (2)	0.082 (2)	0.105 (3)	−0.009 (2)	−0.013 (2)	−0.014 (2)
C1	0.084 (3)	0.0426 (19)	0.087 (3)	0.000 (2)	0.008 (2)	0.0062 (18)
C3	0.061 (2)	0.061 (2)	0.092 (3)	0.010 (2)	−0.001 (2)	−0.001 (2)
C12	0.064 (2)	0.050 (2)	0.080 (3)	0.003 (2)	−0.006 (2)	−0.0140 (19)
C11	0.055 (2)	0.0468 (18)	0.076 (2)	0.0019 (18)	−0.0107 (19)	−0.0103 (17)
C10	0.057 (2)	0.0533 (18)	0.081 (2)	−0.0030 (19)	−0.013 (2)	−0.0078 (18)
C17	0.073 (3)	0.054 (2)	0.099 (3)	−0.004 (2)	0.006 (3)	−0.016 (2)
C18	0.062 (2)	0.076 (2)	0.083 (2)	−0.007 (2)	−0.013 (2)	−0.004 (2)
C4	0.075 (3)	0.084 (3)	0.135 (4)	0.002 (3)	−0.012 (3)	−0.027 (3)
C16	0.090 (3)	0.082 (3)	0.122 (4)	−0.014 (3)	0.029 (3)	−0.017 (3)
C13	0.099 (3)	0.075 (3)	0.086 (3)	−0.009 (3)	−0.010 (3)	−0.009 (2)
C7	0.120 (4)	0.113 (4)	0.086 (3)	0.000 (4)	0.020 (3)	−0.018 (3)
C8	0.065 (3)	0.109 (4)	0.121 (4)	−0.007 (3)	0.015 (3)	0.023 (3)
C15	0.155 (6)	0.097 (4)	0.112 (4)	−0.020 (4)	0.034 (4)	−0.008 (3)
C14	0.150 (5)	0.114 (4)	0.090 (3)	−0.013 (5)	−0.001 (4)	−0.004 (3)
C6	0.148 (6)	0.181 (7)	0.085 (3)	0.015 (6)	−0.009 (4)	−0.037 (4)
C5	0.103 (4)	0.149 (6)	0.130 (5)	0.013 (5)	−0.022 (4)	−0.053 (5)

### Geometric parameters (Å, º)

**Table:**

O1—C9	1.254 (4)	C10—H10B	0.97
C9—N1	1.322 (5)	C17—C16	1.395 (6)
C9—C10	1.506 (5)	C18—H18	0.93
N1—C1	1.451 (5)	C4—C5	1.361 (7)
N1—H1N	0.89 (5)	C4—H4	0.93
C2—C3	1.380 (5)	C16—C15	1.372 (7)
C2—C7	1.380 (6)	C16—H16	0.93
C2—C1	1.529 (5)	C13—C14	1.387 (7)
N2—C17	1.356 (5)	C13—H13	0.93
N2—C18	1.388 (5)	C7—C6	1.396 (8)
N2—H2N	0.96 (6)	C7—H7	0.93
C1—C8	1.509 (6)	C8—H8A	0.96
C1—H1	0.98	C8—H8B	0.96
C3—C4	1.388 (6)	C8—H8C	0.96
C3—H3	0.93	C15—C14	1.377 (8)
C12—C13	1.394 (6)	C15—H15	0.93
C12—C17	1.401 (6)	C14—H14	0.93
C12—C11	1.440 (5)	C6—C5	1.353 (9)
C11—C18	1.357 (5)	C6—H6	0.93
C11—C10	1.491 (5)	C5—H5	0.93
C10—H10A	0.97		
			
O1—C9—N1	121.5 (4)	C16—C17—C12	122.3 (5)
O1—C9—C10	121.2 (4)	C11—C18—N2	110.3 (4)
N1—C9—C10	117.3 (3)	C11—C18—H18	124.9
C9—N1—C1	125.8 (3)	N2—C18—H18	124.9
C9—N1—H1N	116 (3)	C5—C4—C3	120.4 (6)
C1—N1—H1N	118 (3)	C5—C4—H4	119.8
C3—C2—C7	118.4 (4)	C3—C4—H4	119.8
C3—C2—C1	122.6 (4)	C15—C16—C17	117.1 (5)
C7—C2—C1	118.9 (4)	C15—C16—H16	121.5
C17—N2—C18	108.5 (3)	C17—C16—H16	121.5
C17—N2—H2N	136 (3)	C14—C13—C12	118.2 (5)
C18—N2—H2N	115 (3)	C14—C13—H13	120.9
N1—C1—C8	109.0 (4)	C12—C13—H13	120.9
N1—C1—C2	112.4 (3)	C2—C7—C6	119.4 (5)
C8—C1—C2	113.1 (4)	C2—C7—H7	120.3
N1—C1—H1	107.4	C6—C7—H7	120.3
C8—C1—H1	107.4	C1—C8—H8A	109.5
C2—C1—H1	107.4	C1—C8—H8B	109.5
C2—C3—C4	120.9 (4)	H8A—C8—H8B	109.5
C2—C3—H3	119.5	C1—C8—H8C	109.5
C4—C3—H3	119.5	H8A—C8—H8C	109.5
C13—C12—C17	119.1 (4)	H8B—C8—H8C	109.5
C13—C12—C11	133.6 (4)	C16—C15—C14	121.7 (6)
C17—C12—C11	107.3 (3)	C16—C15—H15	119.2
C18—C11—C12	105.8 (3)	C14—C15—H15	119.2
C18—C11—C10	129.2 (4)	C15—C14—C13	121.6 (6)
C12—C11—C10	125.0 (3)	C15—C14—H14	119.2
C11—C10—C9	113.7 (3)	C13—C14—H14	119.2
C11—C10—H10A	108.8	C5—C6—C7	121.8 (6)
C9—C10—H10A	108.8	C5—C6—H6	119.1
C11—C10—H10B	108.8	C7—C6—H6	119.1
C9—C10—H10B	108.8	C6—C5—C4	119.1 (6)
H10A—C10—H10B	107.7	C6—C5—H5	120.5
N2—C17—C16	129.6 (4)	C4—C5—H5	120.5
N2—C17—C12	108.1 (4)		
			
O1—C9—N1—C1	0.1 (5)	C11—C12—C17—N2	−0.5 (4)
C10—C9—N1—C1	179.8 (3)	C13—C12—C17—C16	−1.0 (6)
C9—N1—C1—C8	141.5 (4)	C11—C12—C17—C16	178.3 (4)
C9—N1—C1—C2	−92.3 (4)	C12—C11—C18—N2	0.2 (4)
C3—C2—C1—N1	−6.3 (5)	C10—C11—C18—N2	178.6 (3)
C7—C2—C1—N1	176.6 (4)	C17—N2—C18—C11	−0.6 (5)
C3—C2—C1—C8	117.6 (4)	C2—C3—C4—C5	−0.5 (7)
C7—C2—C1—C8	−59.5 (5)	N2—C17—C16—C15	179.1 (4)
C7—C2—C3—C4	0.2 (6)	C12—C17—C16—C15	0.5 (7)
C1—C2—C3—C4	−176.9 (4)	C17—C12—C13—C14	0.5 (6)
C13—C12—C11—C18	179.4 (4)	C11—C12—C13—C14	−178.7 (4)
C17—C12—C11—C18	0.2 (4)	C3—C2—C7—C6	0.2 (7)
C13—C12—C11—C10	1.0 (6)	C1—C2—C7—C6	177.4 (5)
C17—C12—C11—C10	−178.3 (3)	C17—C16—C15—C14	0.5 (8)
C18—C11—C10—C9	2.6 (5)	C16—C15—C14—C13	−1.0 (9)
C12—C11—C10—C9	−179.3 (3)	C12—C13—C14—C15	0.5 (8)
O1—C9—C10—C11	77.6 (4)	C2—C7—C6—C5	−0.3 (9)
N1—C9—C10—C11	−102.1 (4)	C7—C6—C5—C4	0.0 (10)
C18—N2—C17—C16	−178.1 (4)	C3—C4—C5—C6	0.4 (9)
C18—N2—C17—C12	0.7 (4)	C11—C10—C9—N1	−102.1 (4)
C13—C12—C17—N2	−179.9 (4)	C2—C1—N1—C9	−92.3 (4)

### Hydrogen-bond geometry (Å, º)

**Table:**

*D*—H···*A*	*D*—H	H···*A*	*D*···*A*	*D*—H···*A*
N1—H1*N*···O1^i^	0.89 (5)	2.03 (5)	2.891 (4)	163 (4)
N2—H2*N*···O1^ii^	0.96 (6)	1.91 (6)	2.847 (5)	164 (4)

Symmetry codes: (i) −*x*+1, *y*−1/2, −*z*+3/2; (ii) −*x*, *y*−1/2, −*z*+3/2.

## References

[bb1] Chiou, W.-H., Lin, G.-H., Hsu, C.-C., Chaterpaul, S. & Ojima, I. (2009). *Org. Lett.* **11**, 2659–2662. 10.1021/ol900702t 19456145

[bb2] Farrugia, L. J. (1997). *J. Appl. Cryst.* **30**, 565.

[bb3] Kochanowska-Karamyan, A. J. & Hamann, M. T. (2010). *Chem. Rev.* **110**, 4489–4497. 10.1021/cr900211p PMC2922063 20380420

[bb4] North, A. C. T., Phillips, D. C. & Mathews, F. S. (1968). *Acta Cryst.* A**24**, 351–359.

[bb5] Sheldrick, G. M. (2008). *Acta Cryst.* A**64**, 112–122. 10.1107/S0108767307043930 18156677

[bb6] Siemens (1994). *XSCANS* Siemens Analytical X-ray Instruments Inc., Madison, Wisconsin, USA.

[bb7] Suárez-Castillo, O. R., Sánchez-Zavala, M., Meléndez-Rodríguez, M., Castelán-Duarte, L. E., Morales-Ríos, M. S. & Joseph-Nathan, P. (2006). *Tetrahedron*, **62**, 3040–3051.

